# Prioritizing health information for national health reporting - a Delphi study of the Joint Action on Health Information (InfAct)

**DOI:** 10.1186/s13690-021-00760-8

**Published:** 2022-01-11

**Authors:** Angela Fehr, Stefanie Seeling, Anselm Hornbacher, Martin Thißen, Petronille Bogaert, Marie Delnord, Ronan A. Lyons, Mariken J. Tijhuis, Peter Achterberg, Thomas Ziese

**Affiliations:** 1 ZIG 1 – Information Centre for International Health Protection (INIG), Centre for International Health Protection (ZIG), Robert Koch Institute, Nordufer 20, 13353 Berlin, Germany; 2 Unit 24 – Health Reporting, Department of Epidemiology and Health Monitoring, Robert Koch Institute, General-Pape-Str. 62-66, 12101 Berlin, Germany; 3 Departement of Epidemiology and Public Health, Sciensano, Rue Juliette Wytsmanstraat 14, 1050 Brussels, Belgium; 4 Population Data Science, Swansea University, Singleton Park, Swansea, SA2 8PP UK; 5 Centre for Health Knowledge Integration, National Institute for Public Health and the Environment (RIVM), Antonie van Leeuwenhoeklaan 9, 3721 MA Bilthoven, The Netherlands

**Keywords:** Health information, Health information inequalities, Health information systems, Strategy, Prioritization, Delphi

## Abstract

**Background:**

Health information (HI) strategies exist in several EU Member States, however, they mainly focus on technical issues and improving governance rather than on content-related priority setting. There is also little research available about national prioritization processes underlying HI development for policy support in the EU. The aim of this study was to broaden the knowledge base on HI prioritization strategies and to encourage expert exchange towards good practice models. A specific focus was put on HI produced for national health reporting, this being a crucial tool for policy advice***.***

**Methods:**

We conducted a literature search to identify published and grey literature on national HI prioritization. This was followed by a two-round Policy Delphi study, where we explored which processes and methods exist in EU Member States and associated countries for the prioritization of HI collection. In the first round, information about these processes was gathered in semi-structured questions; in the second round, participants were asked to rank the identified approaches for desirability and feasibility. The survey was conducted online; participants were recruited from the membership of the Joint Action on Health Information (InfAct – Information for Action).

**Results:**

119 experts were contacted, representing 40 InfAct partner institutions in 28 EU Member States and associated countries. Of these, 28 experts responded fully or partially to the first round, and six to the second round. In the first round, more than half of the respondents reported the existence of structured HI prioritization processes in their countries. To prioritize HI, a clear preference was given in the second round for a formal, horizontal process which includes different experts and stakeholders. National public health institutes were named desirable key stakeholders in this process, and also desirable and feasible coordinators for stakeholder coordination.

**Conclusion:**

Health information prioritization methods and procedures reflect the heterogeneity of national public health systems in European countries. Mapping, sharing and ranking prioritization methods and procedures for “good practices” provides a meaningful basis for expert knowledge exchange on HI development. We recommend to make this process part of a future sustainable EU health information system and to use the information gathered in this project to initiate the development of a guidance “Good Practice HI Prioritization” among EU Member States and associated countries.

## Background

Health information inequalities are often linked to health inequalities. Zeitlin defines health information inequalities as “[ …] an unequal capacity to monitor and evaluate population health and health system performance using routinely collected data [ …]” ([1] p. 201). Lack of information about health system performance and population health, as well as untimely or irrelevant information, hinders proper response of health systems to population needs. Or, as Sir Michael Marmot underlined: “All too commonly where health is poorest, health information tends to be poorest” ([2] p. 2).

While plenty of literature exists on health research prioritization, very little is available on health information prioritization in Europe [3]. Our study aimed to contribute to closing this gap. It focused on health information development at national level, specifically on methods and procedures applied to prioritize health information for national health reporting.

Health information (HI) is based on a broad variety of data, e. g. survey or administrative data, on their often secondary analysis and translation into products of health reporting. HI products are mainly directed at scientific communities, political decision makers and the general public, thereby informing research, guiding policies and aiming to shape health behavior at population level [4]. HI facilitates the development and evaluation of targeted and needs-based measures to promote health, prevent disease and deliver health care. It is thus an essential component of the public health action cycle [5, 6]. HI can steer health (in all) policies from two angles: It can be used to evaluate policies and intervention and thus supports an existing agenda (agenda-keeping). At the same time, it may point to emerging issues which require new political and/or scientific attention (agenda-setting) [7]. For HI to adequately support population health and reduce health inequalities, it has to fulfill two objectives: It has to inform about priority population health needs, including health determinants, and about the efficiency of responses of the political and the health system to these needs. Consequently, in the development of HI, particular attention must be paid to the selection and prioritization of indicators that will deliver this information. In this prioritization process, countries often have to consider different agendas. Some HI priorities are outside the scope of national prioritization, but stem from international reporting requirements for which data have to be delivered [8]. Furthermore, national HI priorities may be based on a variety of criteria, from burden of disease-calculation to media-induced attention for selected public health topics. National public health institutes, or other institutions fulfilling national reporting requirements, thus have to respond to calls for data from a variety of stakeholders and decision makers.

HI is developed through population health monitoring and health system assessment. Verschuuren et al. underline that “[a]lthough health data is at the core of population health monitoring, monitoring comprises more than the mere collection and analysis of data. Rather, population health monitoring should be seen as a cycle [ …]. Ideally, this cycle starts with a comprehensive health information strategy. Subsequently, data are being collected based on the needs identified in the strategy, [ …]” ([9] p. 5).

A health information strategy that deals with content related choices would thus be the proper tool to prioritize HI topics and, subsequently, to initiate indicator development and data collection, thereby ensuring that data collection is in line with the identified HI needs. However, despite the necessity to embed population health monitoring in a health information strategy, little research and documentation exists of national strategies or structured methodologies for HI prioritization. We therefore conceptualized a study aiming to gather information on processes and methods for prioritization in HI development at national level. To focus our study, we only addressed the prioritization of HI used for national health reporting, being fully aware that, at national, regional or local level, different stakeholders may produce health data for specific target groups or services.

Our study was part of the Joint Action on Health Information (InfAct - Information for Action) and its work package 5 on the status of health information systems in EU Member States (MS) and regions. InfAct [10] was co-funded by the EU from 2018 to 2021. The project included 40 partners in 28 European countries. Its objective was to build a stronger EU health information system infrastructure and to promote cooperation among EU Member States and associated countries (MS/AC) on issues of HI for policy action. By promoting the availability and usability of adequate HI for researchers, policy-makers and the general public, InfAct aimed to improve population health in Europe.

## Methods

The methods we applied included a literature review and a Policy Delphi survey for data collection. A method paper which included the outline for the literature search, the draft questionnaire and accompanying documents for the survey was prepared with experts from six InfAct partner countries [11]. The following paragraphs describe both methods.

### Literature review

A literature search was performed to identify published or grey literature on strategies for national HI prioritization which would inform the development of the questionnaire for the Delphi survey. The literature review was based on the search strategy set out in the report on “Priority setting methods in health information” of a previous project, the BRIDGE Health [3]. Our search strategy took a wider scope, expanding the original BRIDGE search strategy below:

- ((((priorit*[Title]) AND (((set*[Title]) OR determin*[Title]) OR develop*[Title]))) OR ((research [Title]) AND priorit*[Title]))

Our search augmented the original BRIDGE search, by including additional terms for health information prioritization:

- OR ((((health [Title]) AND information [Title]) AND priorit*[title/abstract]) AND ((report [title/abstract]) OR (policy [title/abstract])))

In January 2019, we applied our search in the PubMed and Embase literature databases, and in the OpenGrey grey literature database and limited results to publications within the last ten years. Results obtained from hand search of bibliographies of included studies were also counted in, as well as studies identified as relevant by experts. For terms related to overall prioritization, we limited our search to article titles only. For terms related to HI prioritization we allowed more flexibility by including results from article titles and abstracts. This approach helped limit the number of search hits and focused our search on articles whose main objective was HI prioritization.

Our search returned 5010 articles which focused mostly on overall prioritization methods used at the community or regional level. Articles were de-duplicated, resulting in a total of 2952 articles for review. In order to isolate articles discussing national prioritization processes, we filtered results by selecting articles whose abstract contained the words ‘nation*’. Through a review of the resulting 990 articles’ titles and abstracts we excluded articles that did not outline a prioritization methodology, that were applied among non-human subjects, and articles that did not cover national level prioritization. We reviewed the full text and bibliographies of 182 articles, identifying 111 articles which were relevant for national HI prioritization.

**Fig. 1:**
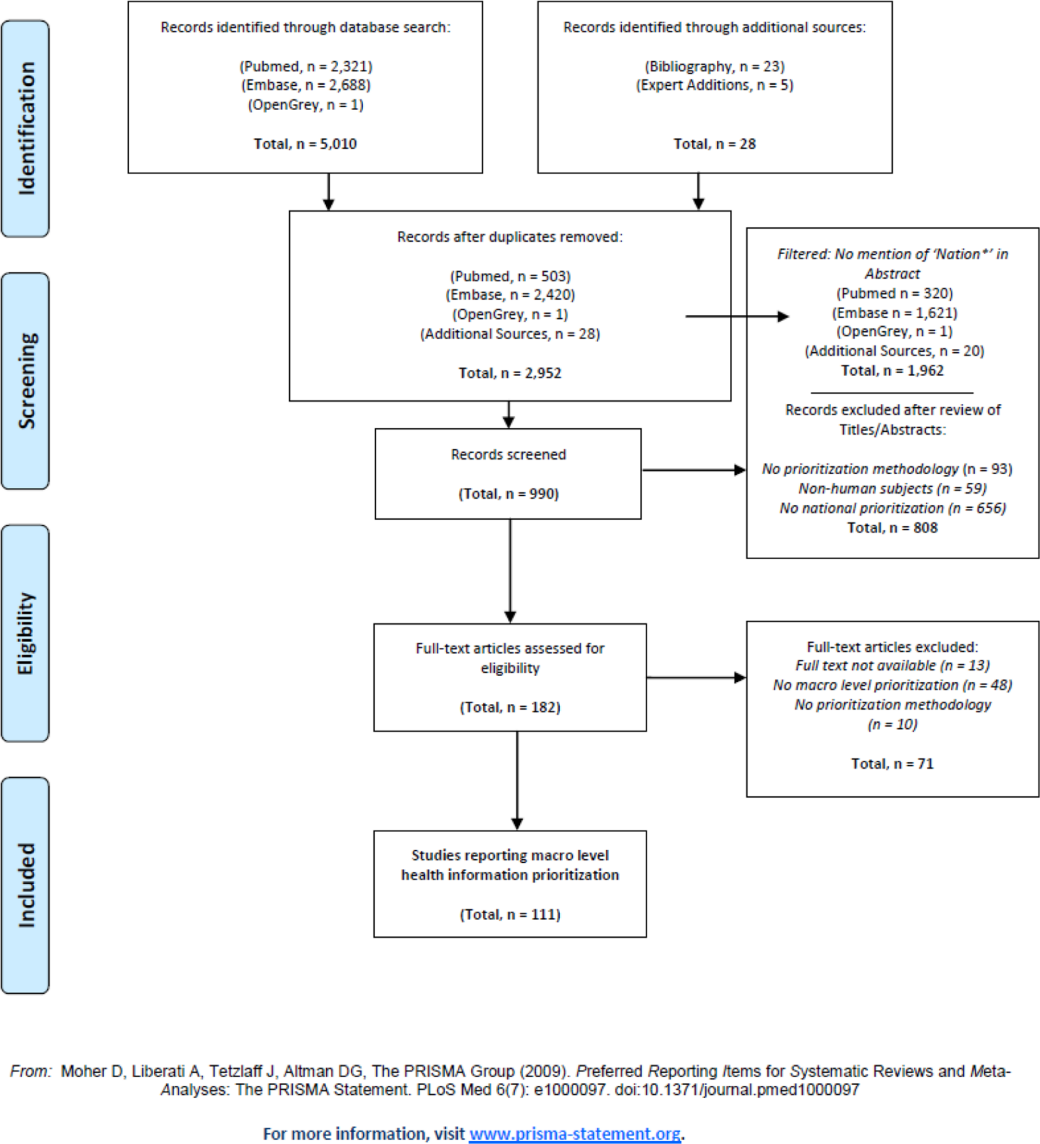
Health Information Prioritization ─ PRISMA flow diagram on the results of the search strategy. Reported following PRISMA statement recommendations [12]

Figure 1 contains a PRISMA diagram [12], outlining the procedure for our literature review.

Out of the initial set of publications, 13% of our findings referred to procedures and examples of priority setting applied by EU MS, whereas 42% of the publications focused on national priority setting procedures for developing and emerging countries. However, examples and frameworks for priority setting, both for developing countries as well as for EU MS, rarely focused on HI. Instead, through the aggregation of our findings to EU MS only, it became apparent that the majority of the European examples focused on the priority setting processes in research or health care. Hence, it was decided to complement the initial search by a semi-systematic search for grey literature on the prioritization of HI. Through the assessment of the grey literature, it became clear that most of the EU MS seem to prioritize and process HI through frameworks of national health reporting, each with its own formats and priorities. Individual MS, e.g. Austria or Germany, set (selected) priorities in HI implicitly through the definition of national health targets and the related procedures of national health reporting.

The preparation, operationalization and realization of health targets through the Rahmengesundheitsziele (health targets framework) in Austria started 2010 with a broad participatory approach. Austrian health targets are based on a number of guiding principles like the focus on health determinants, the “health-in-all-policies approach” or the promotion of health equity. The establishment of Austrian health targets has an indirect impact on health reporting and the prioritization of HI, for instance through the simultaneous integration of the promotion of equal opportunity and social welfare as a health target and a criterion for national health reporting [13].

In Germany, a national health targets process was established in the year 2000. To date, nine health targets have been defined. Topics for potential health targets are selected by a group of experts on the basis of defined criteria, including mortality, morbidity, or health economics. Measurability and the related data availability are further criteria which inform the selection of a topic as national health target. So, while national health targets in Germany depend on data from health monitoring and health reporting to measure change, there is no process established to ensure that the topic selection for national health monitoring activities considers indicators relevant to national health targets [14].

### Data collection through a Delphi process

As method of choice for our study we selected a Policy Delphi survey. As in a classical Delphi survey [15], participation in a Policy Delphi is anonymous, and the survey is conducted in several rounds. Participants are not a numerical sample of a given population of experts, but a sample of available expertise. Purposive sampling is needed for depth and specificity of expertise [16]. Heterogeneity of the panel is considered to be of benefit, as it minimizes the risk of overlooking obvious aspects of a question [17], while homogeneity of the level of expertise is a decisive factor for the validity of a Delphi survey’s outcome [18]. A Policy Delphi differs from a classical Delphi insofar as it does not aim to achieve consensus among participants. Instead, a Policy Delphi gathers a range of options which are ranked according to their degree of “desirability”, “feasibility”, “importance” and “confidence” [19]. Because of this ranking and prioritizing of options, the output of a Policy Delphi survey is in general considered operational for a variety of actors, including policy-makers [20].

Based on the results of the literature review and in close collaboration with experts from InfAct, our study was designed along the following questions:

- How is health information for national health reporting prioritized in EU MS and associated countries?
- Are criteria, methods or structured processes used to prioritize health information at national level?
- Which stakeholders are involved in prioritization processes?
- How is stakeholder involvement organized?
- How is health information linked to health targets and frameworks, both national and international?
- Can good practice approaches in prioritizing health information be identified?

The survey was conducted online in two rounds, using the Voxco Online software. This tool has extensively been used for health monitoring by the Health Survey Lab at the Robert Koch Institute (RKI). Technical support was available in-house for programming and survey implementation. Data protection approval was obtained from from the Data Protection Officer at the RKI as implementing institution. A pre-test was conducted in three countries of the InfAct consortium to identify issues of comprehensibility and technical implementation.

Representatives of the InfAct partner institutions were invited to participate in the survey. For the live survey, 119 potential participants, representing the 40 InfAct partner institutions in 28 EU MS/AC, received an email which included a link to access the survey as well as a letter of invitation, a project summary and information on anonymity and data protection. The study was implemented between September 2019 and May 2020; two reminders were sent before closing each round. To access the questionnaire, potential participants had to give their informed consent. At the end of the first-round questionnaire, participants were asked to indicate whether they are willing to be invited to the second round; only for participants who confirmed their willingness, contact data were securely stored.

The first round of the survey focused on the gathering of information from experts at country level, combining structured, mainly multiple-choice and a few full-text questions. The second round required a ranking of options. Following the first round, qualitative data were analyzed using the text-sorting technique by Beywl & Schepp-Winter [21]. From the full-text replies, categories were developed which formed the basis for the response options in the structured questions for the second round. Categories were developed through iterative reading and identification of similarities and in-vivo-codes; three researchers participated in the development, review and refinement of the categories and the wording of the second questionnaire. The questionnaires of both rounds are published in Fehr et al. (2021) [22].

A specific feature of Delphi surveys with several rounds is the feedback: With each new round, participants receive the quantitative results of the previous round as well as their own replies so that they can re-consider, confirm or alter their responses in the light of the analysis of the previous round. We used the feedback to inform participants in the second round of the quantitative results of the first round in graphic format and included all full-text replies. Owing to data protection, the respondents did not receive their responses from the previous round and the replies were cleaned from any information which would have revealed the respondents’ identity (e.g. mention of country or institution). In the second round, participants were asked to rank questions according to the degree of “desirability”, “feasibility”, “importance” and “confidence” based on the Policy Delphi Survey methodology by Turoff (2002) [19]. As recommended by Turoff, no neutral position was included in the rating scale. Instead, participants were given a fifth option labeled “no judgement”.

## Results

The following paragraphs show the results of the first and the second round of the survey. Replies are not separated by round but are organized in thematic blocks for both rounds. The analysis for the second round focuses on the categories “desirability” and “feasibility”, which were considered to be the most relevant for the research question. To benefit from the total number of responses to individual questions of the survey, frequency distributions include fully as well as partially completed questionnaires, with valid n calculated for each questionnaire item.

### Response

Of the *N* = 119 experts to whom the invitation to the first round of the survey was sent, *n* = 28 started the survey and gave responses, whereby *n* = 17 participants (14%) fully and *n* = 11 (9%) partially completed the questionnaire. 2 (2%) actively denied participation by selecting “no” on the Informed Consent-Page (screened out); in 86 instances, the questionnaire or browser window was closed without responding to questions. 17 participants had given consent to participate in the second round; of these, 6 completed the second questionnaire. No drop-outs or screened out participants were registered by the system for the second round.

Since the survey was conducted under the rule of anonymity, no information on country affiliation of respondents was requested.

### Background and involvement in health information development of respondents

In the first round, participants were asked to provide information on their professional background, their professional affiliation and the degree to which they are involved in HI development. The majority of the respondents (11/26) had a professional background in public health, followed by medicine and epidemiology (7/26) each. Five respondents had a professional background in statistics and 4 in informatics; 3 respondents indicated a background in demography and 2 in political science. For the section on professional backgrounds, multiple answers were possible; additionally, for backgrounds not listed, a full-text field was provided. Here, 7 respondents indicated backgrounds in life sciences, economics, mathematics and social sciences. In the section on professional affiliation, 11 respondents reported an affiliation with a national public health institute, while 5 indicated that they were affiliated with a ministry of health. One participant was affiliated with the national statistics office, and one with the ministry of research. A total of 5 respondents used the full-text-field labeled “other” and added an affiliation with health research, health information, a university or an unspecified institute related to a ministry of health.

In addition to providing a defined professional affiliation and background, the survey participants were asked to self-rate their degree of involvement in HI development. Most of the respondents declared that they are very highly (*n* = 6/23; 26%) or highly (*n* = 10/23; 44%) involved, while 7 respondents chose the options medium (*n* = 4/23; 17%) or low (*n* = 3/23; 13%) involvement, indicating that they are neither in charge of HI development nor represent a key stakeholder, but irregularly act as participant or observer in HI development processes.

### Existence of structured health information prioritization

**Fig. 2:**
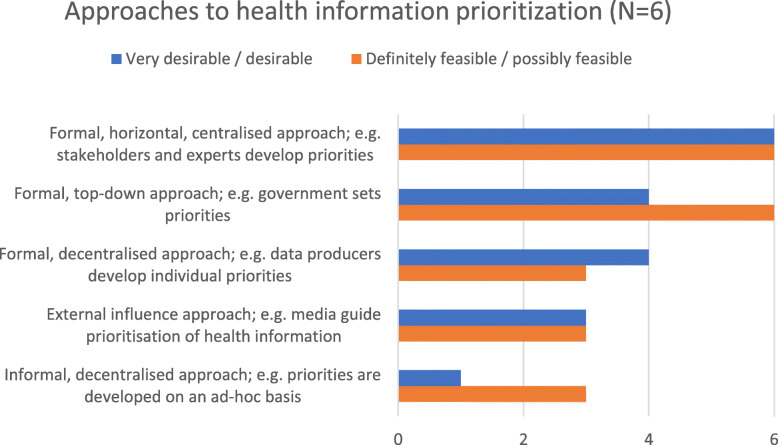
Approaches to health information prioritization

In the first round of the survey, participants were asked whether and which processes for HI prioritization existed in their countries. A little over half (*n* = 15/26; 58%) of the respondents confirmed the existence of structured HI prioritization processes; 73% of these (*n* = 11/15) stated that these processes are documented or published. From the full-text description of the processes provided by respondents, the research team developed five approaches to HI prioritization which participants ranked in the second round. Figure 2 below shows the aggregated positive responses for the categories desirability (very desirable/desirable) and feasibility (definitely feasible/possibly feasible). The formal, horizontal approach, where different experts and stakeholders are included in the prioritization process, received positive ratings from the *n* = 6/6 respondents both for desirability as well as for feasibility. A formal, decentralized approach, where e.g. data producers develop individual priorities, is desirable for *n* = 4/6 and feasible for *n* = 3/6 respondents. The formal, top-down approach, where governments set priorities was considered feasible by all respondents (*n* = 6) and desirable by n = 4/6.

Approaches where external influences, such as the media, guide prioritization, were rated less desirable. The same was true for informal, decentralized approaches, where priorities are developed on an ad-hoc basis. Externally influenced prioritization approaches were also the only option which *n* = 2/6 respondents rated definitely unfeasible.

### Stakeholder involvement in health information prioritization

**Fig. 3:**
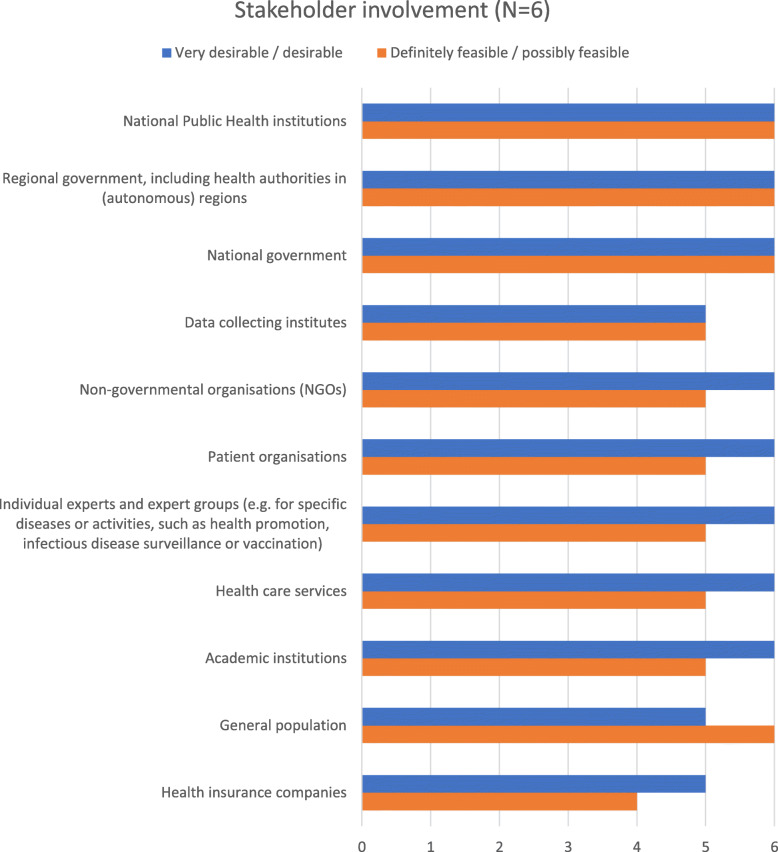
Stakeholder involvement

A crucial question in prioritization for HI development is the involvement of stakeholders. Participants were therefore asked whether stakeholders are involved in such processes in their countries, which stakeholders are involved and who takes over a coordinating function for stakeholder participation. About two thirds of the respondents in the first round (*n* = 17/26; 65%) confirmed that stakeholders are involved in HI prioritization processes. Respondents added a list of stakeholders which are involved in their country and stated who coordinated stakeholder involvement. Based on this list, participants in the second round were asked which stakeholders should be involved in prioritization. All respondents considered involvement of policy-makers (national or regional governments) and national public health institutes positively in terms of desirability and feasibility. Of the remaining options only one (general population) was considered more feasible than desirable to involve as stakeholder (Fig. 3).

Assuming that effective stakeholder involvement needs coordination, participants were asked in the second round whom they considered desirable and feasible for this coordinating function. Based on an aggregation of the positive replies, most of the respondents selected national public health institutes (*n* = 6/6) and national governments (*n* = 5/6) for both categories. Few respondents considered it desirable or feasible to hand over stakeholder coordination to patient organizations, non-governmental organizations (NGOs), health insurance companies, the general population, or health care services.

### Frameworks guiding health information prioritization

National governments must fulfill data reporting obligations vis à vis international and regional organizations, often through their public health institutions or their national statistical offices. We proceeded from the assumption that prioritization of HI is therefore partly guided by national or international frameworks or methodologies. At the same time, and irrespective of reporting obligations, national, regional or international action plans, or comparable documents guiding health policy, may be a source of priorities, or criteria used in national prioritization processes for HI. In the first round, and in response to the question whether criteria are used in prioritization, *n* = 14/26 of the participants replied affirmatively; within this group, *n* = 13/14 stated that these criteria are linked to national or international frameworks or methodologies, in particular to national health strategies (*n* = 11/13) and national health targets (*n* = 10/13). Other frameworks included the Sustainable Development Goals (SDGs) (*n* = 6/13), national burden of disease studies and WHO Action Plans (*n* = 5/13), Global Burden of Disease Studies (*n* = 4/13) or, specifically, the Framework Convention on Tobacco Control (*n* = 4/13).

In the second round, participants were asked which frameworks, from the point of view of their country, should guide HI prioritization. All listed frameworks or methodologies were considered either very desirable or desirable and definitely feasible or possibly feasible guiding instruments; one participant stated that it was possibly unfeasible to use Global Burden of Disease Studies to guide prioritization.

### Criteria development for health information prioritization

It was also of interest to learn who is involved in criteria development for HI prioritization and which are the preferred actors. To this end, the participants were asked to rank a list of 12 actors according to desirability and feasibility. In this section, aggregate counts for positive replies (very desirable/desirable; definitely feasible/possibly feasible) were calculated. As shown in Table 1 and Table 2 below, national public health institutes were seen as a desirable and feasible actor by all respondents (*n* = 5/5). Regional governments, including health authorities in (autonomous) regions, health care services, national governments and national statistical offices received *n* = 4/5 positive replies regarding desirability. As regards feasibility of involvement, regional governments were considered a feasible actor by all respondents (n = 5/5) and national governments by most of the respondents (*n* = 4/5). The results for the remaining actors on the list showed more variance. To illustrate, health insurance companies received a quite balanced voting, i.e. *n* = 2/5 each positive and negative replies for desirability and *n* = 2/5 positive and *n* = 3/5 negative votes for feasibility. Patient organizations were rated mainly positively for desirability (*n* = 3/5), but received balanced positive and negative ratings (*n* = 3/6 each) for feasibility; this was the only question in the section which received 6 instead of 5 replies. Health care services were considered very desirable/desirable stakeholders in criteria development (*n* = 4/5), but only 2 participants considered their involvement definitely or possibly feasible. The outcome for NGOs was mainly positive for desirability (*n* = 3/5), but only 1 participant rated their involvement definitely or possibly feasible. Participants’ views also differed on a broad participatory approach, i.e. involving the general population in criteria development for HI prioritization; only 1 participant considered it a definitely or possibly feasible option while *n* = 2/5 considered it very desirable/desirable. Opinions were equally split on data collecting institutes, whose involvement was considered very desirable/desirable by *n* = 3/5, while for 1 respondent their involvement was very undesirable – indeed the only actor in the list that received this negative ranking. In terms of feasibility, the majority (*n* = 4/5) considered the involvement of data collecting institutes to be feasible. Of note, in this block auf questions, for the first time in this study respondents repeatedly selected the option “no judgement”.

**Table 1:** Desirability of involving actors in criteria development for health information prioritization

Desirability					
	Very desirable / desirable	Undesirable	Very undesirable	No judgement	Total
National Public Health institutions	5	0	0	0	5
Regional government, including health authorities in (autonomous) regions	4	0	0	1	5
Health care services	4	0	0	1	5
National government	4	0	0	1	5
National Statistical offices	4	0	0	1	5
Data collecting institutes	3	0	1	1	5
Non-governmental organizations (NGOs)	3	1	0	1	5
Academic institutions	3	2	0	0	5
Patient organizations	3	1	0	1	5
Individual experts and expert groups (e.g. for specific diseases or activities, such as health promotion, infectious disease surveillance or vaccination)	2	2	0	1	5
General population	2	1	0	2	5
Health insurance companies	2	2	0	1	5

**Table 2:** Feasibility of involving actors in criteria development for health information prioritization

Feasibility					
	Definitely feasible / possibly feasible	Possibly unfeasible	Definitely unfeasible	No judgement	Total
Regional government, including health authorities in (autonomous) regions	5	0	0	0	5
National Public Health institutions	5	0	0	0	5
Data collecting institutes	4	1	0	0	5
National government	4	1	0	0	5
National Statistical offices	4	0	0	0	4
Individual experts and expert groups (e.g. for specific diseases or activities, such as health promotion, infectious disease surveillance or vaccination)	3	2	0	0	5
Academic institutions	3	2	0	0	5
Patient organizations	3	2	1	0	6
Health care services	2	2	1	0	5
Health insurance companies	2	3	0	0	5
General population	1	3	1	0	5
Non-governmental organizations (NGOs)	1	3	1	0	5

All respondents (*n* = 6/6) agreed that it is desirable and feasible to use criteria lists and perform literature and document searches for the criteria development process. Equally, respondents considered it very desirable/desirable to have meetings of mixed groups consisting of researchers and policy-makers; the majority also saw these as definitely or possibly feasible. One respondent ranked them possibly unfeasible. Of interest, while surveys, expert consultation via email and expert face-to-face meetings were regarded as feasible options, they were not considered desirable by all respondents. Two respondents declared that expert consultation via email was undesirable; web-based public consultation, data analyses and expert face-to-facemeetings were also rated undesirable by one participant, each.

### Good practice approaches

The questionnaire for the first round concluded with the question which, if any, national efforts existed in participants’ countries to develop national good practices for prioritization of HI. From all full-text replies, the following ten approaches (Table 3) were summarized for participants to rank in the second round:

**Table 3:** Good practice approaches

i. Implement a national health information strategy	
ii. Implement national health targets	
iii. Implement a national legal act on health information (covering e.g. data standards, health information systems, e-health, infrastructure)	
iv. Set up a national catalogue on health information (including e.g. organisation, processes and standards around health care and health indicators)	
v. Develop guidelines at ministerial level (covering e.g. prevention, diagnostics and therapy)	
vi. Develop guidelines at intersectoral / institutional level for health information and / or health reporting	
vii. Develop topic/disease-specific good practices for health information and indicator development	
viii. Scale up topic/disease-specific good practices for health information and indicator development	
ix. Develop data quality frameworks	
x. Establish “unique health identifiers”	

The majority of these approaches were considered either very desirable or desirable by the *N* = 5 respondents. The preferred approach (*n* = 4/5 very desirable, *n* = 1/5 desirable) was the implementation of a national health information strategy (Table 3 item i.). Only one approach, i.e. to implement a HI law (item iii.), was considered undesirable by one participant. One participant opted for “no judgement” on item x., i.e. the unique health identifiers.

**Fig. 4:**
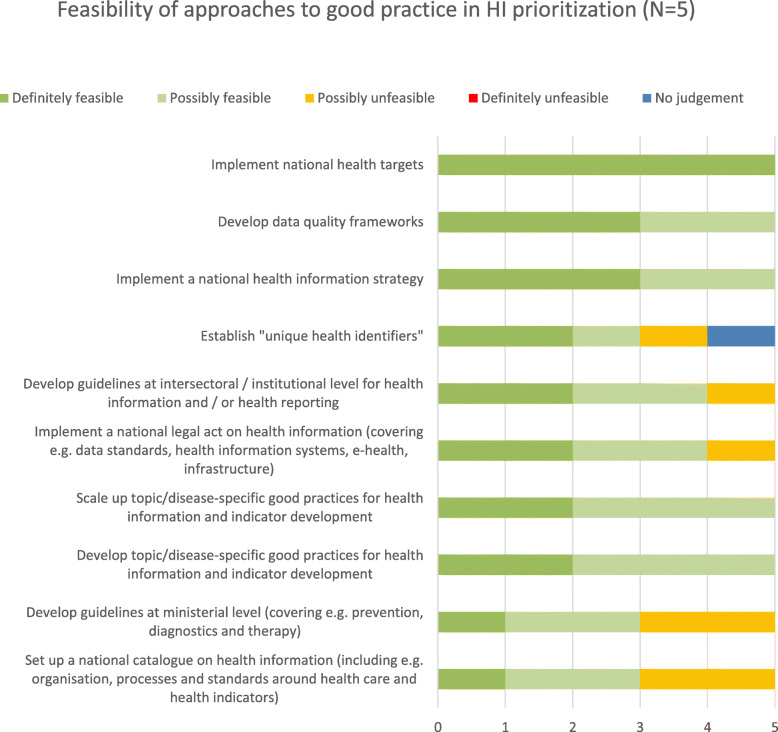
Feasibility of approaches to good practice in health information prioritization

Ranking for feasibility of these approaches was slightly more diverse (Fig. 4). All respondents (*N* = 5) considered the implementation of national health targets definitely feasible. In total, five approaches received aggregated positive rankings while the remaining five were rated more critical.

**Fig. 5:**
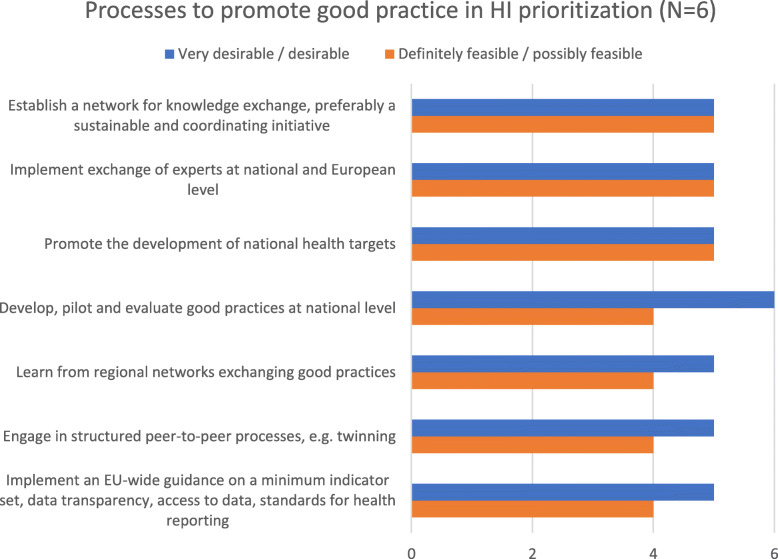
Processes to promote good practice in health information prioritization

When asked for processes to promote good practice in HI prioritization at national level, participants considered developing, piloting and evaluating good practice approaches to be very desirable or desirable. Promoting the development of health targets as well as networking and expert exchange, with a coordinating component and at national and European level, were seen as desirable as well as feasible approaches. Equally desirable were to implement an EU-wide guidance on a minimum indicator set, data transparency, access to data, standards for health reporting; to engage in structured peer-to-peer processes, e.g. twinning; or to learn from regional networks exchanging good practices. One participant each rated these options possibly unfeasible (Fig. 5).

### Improving health information systems for users and policy-makers

Since health information systems are an essential component of health systems, participants were encouraged to indicate which improvements to their national HI systems would benefit HI users and in particular policy-makers. As shown in Table 4 below, the full-text answers for suggested improvement, which assumingly reflect the current status of the national HI system of the respondent, touch upon all areas of HI, from establishing legal frameworks to selecting indicators for health in all policies (HiAP) and public health needs to process coordination and developing adequate formats for HI dissemination.

**Table 4:** Improvements to national health information systems

Which improvements to your national health information system would benefit health information users and policy makers in your country? *Improvements may include continual indicator alignment with population health needs, data quality improvements, or improvements in dissemination (including access or formats used).*	
**Responses**	
The largest improvement has been the legislation […]. The threat is that GDPR is interpreted stricter than national legislation in the future.	
Clear mandate to coordinate.	
Indicators should build a complex picture about health status/needs/issues of citizens, including also public and environmental health.	
Data quality improvements and more use of linked data o report on outcomes.	
Complete redefinition of indicators and collection/reporting methodology.	
Implementation of a unique identifier for health.	
Alignment with population health needs. Improvement in public health dissemination of data improvement in public health communication.	
Improvements in dissemination including the format of interpretation responsive for various levels (public, patients, health care providers, payers, policy).	
Integration of more interests.	
Introduce legislation regarding data collection, analysis and process.	
Continual indicator alignment and also minimum requirements on data transparency and openness (also for third-party research) are likely to improve our health system development in my country.	
At present there is no published health information strategy […]. This is being prepared and it is hoped that this will benefit producers and users of health information. It is hoped that such strategy will help to make clear the role of the various stakeholders/producers of health information.	
The information provision process is under review as to find new ways on conveying Information (e.g., more easy-to-read “fact Sheets” and less large scale overall reports). […] strict data protection regulations leading to highly fragmented data ownership and management combined with a federalistic governance structure in administrations, i.e. a lot of information cannot be combined on person basis (from the spheres of sickness funds, Province owned Hospitals and data owned by the State such as social-demographic data like income).	
More emphasis on policy analysis. More emphasis on policies outside the health domain and their effect on health.	
The health information system strategy will look to address issues such as coverage, gaps, data linkage and health information capacity more generally in order to benefit system users and policy makers.	
Modernizing of web-based presentation of indicators.	

GDPR = General Data Protection Regulation

## Discussion

The Policy Delphi proved to be a suitable instrument for our research question. Participants in the first round readily shared information on strategies for HI prioritization, and the ranking of options from these responses for their degree of desirability and feasibility may guide next steps towards a draft guidance on HI prioritization. For the research question, these two categories were of particular relevance, while the categories “importance” and “confidence” could become part of a more detailed analysis at a later date.

Through the InfAct network, we approached 119 experts which represented 40 institutions in 28 countries. For reason of data protection, we cannot determine whether each of the 28 participants in the first round represents one country or one institution, or whether some countries were represented by more than one respondent. The 17 participants in the first round, who agreed to receive an invitation for the second round, represented 14 countries. Again, for reason of anonymity, we cannot say whether the six participants to the second round represented six different countries. We had indeed hoped for a higher response and are fully aware of the limitations which result from the low number of survey participants. Still, we are very grateful to all experts who devoted time to share their knowledge to advance the discussion around objectives and methods of HI prioritization. From the volume of contributions which we received we conclude that the topic met with interest in the InfAct community, and that the study fulfilled its aim to establish a knowledge base on HI prioritization to be developed and used for further expert exchange. We are particularly grateful to the participants of the second round of the survey, since its implementation from March to May 2020 coincided with the SARS-CoV-2 outbreak and its extraordinary burden on the public health community, also in the InfAct network.

We found that literature on prioritization often discusses health research or prioritizing resources in health care. If it focuses on HI, it is rather concerned with the development of priorities for (new) health indicators or for indicator sets based on existing data and not so much with prioritizing topics for strategic HI development. Against this background, our study filled a gap by compiling information on strategic national approaches and structured methods for HI prioritization, and by engaging HI experts in the identification of good practice models. The survey identified that a majority of participating countries apply structured processes to HI prioritization. The national processes differed considerably. They ranged from ad-hoc approaches, where new indicators are sometimes developed following frequent requests from stakeholders, media or the research community, over decentralized processes, where different data collecting institutes have their own processes and decision-making on data collection, to stringent and centralized processes where ministries of health determine HI priorities. Respondents to our survey indicated that ad-hoc approaches to selecting topics is not a desirable option. Wider preference was given to structured approaches, such as a health information strategy or health targets, either coordinated by ministries or enabling cooperation among experts and stakeholders more horizontally.

The majority of respondents indicated that different stakeholders are involved in HI prioritization. Suggestions for stakeholder involvement ranged from national public health institutes to the general public. Some stakeholders, e.g. regional governments, were deemed desirable partners in HI prioritization on the one hand, but some respondents doubted the feasibility of their involvement. Here, potential roadblocks could be explored, and ideally removed, to increase feasibility of desired involvement. Also, further discussion could be of interest to identify perceived hindrances to the desirability and feasibility of selected stakeholders’ involvement in criteria development.

To promote science-base, transparency and comprehension of HI prioritization, we would recommend that networks, such as the National Nodes on HI established under InfAct [23], be used to collaborate in drafting a guidance for the development of a health information strategy or a guidance for “Good Practice HI Prioritization”. Such documents could contain cornerstones for HI prioritization while being adaptable to the context of national health information systems, building on international guidances on priority setting in health. A model health information strategy could function as a sustainable national framework for HI prioritization, which connects processes such as national health targets, national health monitoring and national public health strategies for mutual benefit and support. A guidance “Good practice HI prioritization” could complement the existing “Good Practice Health Reporting” [24] at the very opposite end of the HI development process (i.e. from HI strategy to HI product). Possible topics to be covered by a guidance on HI prioritization could be criteria for HI prioritization, including burden of disease, data availability, reporting obligations, national or international health policy agendas, actionability, social or economic impacts as well as stakeholders in HI prioritization, including lead/coordination of stakeholders, selection and procedures for involvement of stakeholders (considering conflicts of interest), degree of involvement.

Proceeding from national guidances, it may be worthwhile to explore the development of a European framework for HI prioritization with a view to facilitating decision-making processes for pan-European data collections.

Several participants in the survey indicated that structured prioritization processes in their country are documented in health reports or publications. To follow-up on our study, these documents should be reviewed as source documents for a potential guidance on HI prioritization or on a model health information strategy. Countries whose HI prioritization processes are documented as part of national health reports, or published, should also be encouraged to include them in the newly launched Health Information Portal [25] for easy reference for the European public health community. The Health Information Portal would also be a suitable location to disseminate a guidance on “Good practice HI prioritization”.

## Conclusions

Health systems in Europe are heterogeneous, and so are health information systems. Prioritization methods and procedures for national health reporting are embedded in national public health systems, reflecting this heterogeneity. No singular approach to prioritization can be considered best practice. We are convinced that a mapping, sharing and ranking of prioritization methods and procedures for good practices provides a meaningful basis for expert knowledge exchange on HI development in the context of building a sustainable EU health information system. We are hopeful that this study will contribute to national and European processes aimed at transparent and comprehensible selection and prioritization of HI for the benefit of adequate provision of health promotion, prevention and care.

## Data Availability

Please contact the corresponding author for data requests.

## Abbreviations

EU: European Union
EU MS/AC: EU Member States and Associated Countries
GDPR: General Data Protection Regulation
HI: Health information
HiAP: Health in all policies
InfAct: Joint Action on Health Information (Information for Action)
MS: Member States
NGOs: Non-governmental organizations
RKI: Robert Koch Institute
SDGs: Sustainable Development Goals
